# Rapid Diagnostic Testing in Bloodstream Infections: A Retrospective Clinical and Economic Evaluation from a University Hospital in Bulgaria

**DOI:** 10.3390/microorganisms14030675

**Published:** 2026-03-16

**Authors:** Ralitsa Raycheva, Gergana Lengerova, Michael Petrov, Todor Kantardjiev

**Affiliations:** 1 Department of Social Medicine and Public Health, Medical University Plovdiv, 15A V. Aprilov Blvd., 4002 Plovdiv, Bulgaria; r.raycheva@mu-plovdiv.bg; 2 Department of Medical Microbiology and Immunology “Prof. Dr. Elissay Yanev”, Medical University Plovdiv, 15A V. Aprilov Blvd., 4002 Plovdiv, Bulgaria; michael.petrov@mu-plovdiv.bg (M.P.);; 3 Laboratory of Microbiology, University Hospital St. George, 15A V. Aprilov Blvd., 4002 Plovdiv, Bulgaria; 4 Research Institute at Medical University Plovdiv, 15A V. Aprilov Blvd., 4002 Plovdiv, Bulgaria

**Keywords:** bloodstream infections, rapid diagnostic tests, antimicrobial stewardship, cost-effectiveness, hospital length of stay, antibiotic optimization, health economic evaluation, intensive care unit

## Abstract

Rapid diagnostic tests enable earlier pathogen identification in bloodstream infections compared with conventional culture-based methods and may improve clinical and economic outcomes, particularly when integrated with antimicrobial stewardship programs. Evidence suggests that while mortality benefits are context-dependent, rapid diagnostics can optimize antibiotic use and hospital resource allocation. The present study aimed to evaluate the clinical and economic impact of rapid diagnostic approaches compared with conventional microbiological culture in patients with confirmed bacteremia or fungemia hospitalized in a tertiary care setting in Bulgaria. A retrospective observational study was conducted between January 2015 and August 2020 at University Hospital “St. George,” Plovdiv. A total of 115 patients with confirmed bacteremia or fungemia were included and allocated to either a rapid diagnostic testing group (*n* = 77) or a standard culture group (*n* = 38). Mortality rates were comparable between groups (54.5% vs. 55.3%; OR 0.97, 95% CI 0.45–2.12; *p* = 0.942). Median length of stay was 20 days (12–35) in the rapid-test group versus 16 days (10–31) in the culture group (*p* = 0.505). Targeted antibiotic therapy duration was longer in the rapid-test group (median 12 vs. 6 days; *p* = 0.070). Median direct hospital costs were BGN 2319.40 versus BGN 1855.52, and indirect costs were BGN 19,388.80 versus BGN 15,511.04 (both *p* = 0.505). Diagnostic costs were significantly higher in the rapid-testing group (BGN 55.00 vs. BGN 38.00; *p* = 0.002). Rapid diagnostic testing produced clinical outcomes comparable to standard culture while demonstrating context-dependent economic differences in hospital resource utilization. Conclusions: Rapid diagnostic testing for bloodstream infections provides clinical outcomes comparable to standard culture-based methods while offering potential economic differences associated with the diagnostic strategy. When combined with antimicrobial stewardship interventions, rapid diagnostics support optimized antibiotic use and more efficient hospital resource utilization in critically ill patients.

## 1. Introduction

Rapid diagnostic approaches for bloodstream infections (BSIs) have been proposed as a cost-effective strategy in hospital care, particularly when combined with antimicrobial stewardship programs. Multiple systematic reviews and meta-analyses demonstrate that molecular rapid diagnostic tests (mRDTs) and other rapid platforms (e.g., MALDI-TOF, multiplex PCR) significantly reduce time to targeted therapy, hospital length of stay, and mortality compared to conventional culture-based methods, with the greatest benefit observed when results are actively communicated to clinical teams and stewardship interventions are in place [1,2,3,4,5].

Economic modeling shows that strategies such as MALDI-TOF with stewardship can result in substantial cost savings per quality-adjusted life year and prevent additional deaths compared to standard laboratory methods. The cost-effectiveness is most pronounced when rapid diagnostics are integrated with stewardship, as isolated implementation of rapid tests without stewardship yields less consistent clinical and economic benefit [1,3,5].

The American Society for Microbiology, in its 2025 evidence-based laboratory medicine guidelines, strongly recommends the use of rapid diagnostic tests in conjunction with active communication to decrease time to targeted therapy and hospital stay, supporting their utility in improving patient outcomes and resource utilization [2]. Randomized trials confirm that rapid identification and susceptibility testing lead to faster antibiotic modifications, although direct improvements in patient outcomes may depend on the clinical context and stewardship engagement [6]. A growing body of literature suggests that rapid diagnostics for bloodstream infections, particularly when combined with antimicrobial stewardship programs, may improve timeliness of care and antimicrobial optimization, with potential downstream effects on clinical outcomes and hospital resource utilization [1,2,3,4,5,6].

Despite substantial international evidence supporting rapid diagnostics combined with antimicrobial stewardship, important gaps remain. Most cost-effectiveness data derive from multicenter trials or modeling studies conducted in Western healthcare systems with established stewardship infrastructures [1,3,7,8]. Real-world implementation in resource-variable tertiary hospitals, particularly in Central–Eastern Europe, has been less extensively studied [7,9,10,11,12,13]. The European Society of Clinical Microbiology and Infectious Diseases specifically notes that recommendations for antimicrobial stewardship and rapid diagnostics must be adapted to local settings, given the scarcity of high-quality studies and the variability in evidence certainty [14]. Differences in hospital financing structures, antimicrobial prescribing patterns, and workflow integration may substantially influence both clinical and economic outcomes. Therefore, context-specific evaluations are necessary to determine whether the benefits observed in controlled settings translate into routine clinical practice.

The present study therefore aimed to evaluate the clinical and economic impact of rapid diagnostic approaches compared with conventional microbiological culture in patients with confirmed bacteremia or fungemia hospitalized in a tertiary care setting in Bulgaria.

## 2. Materials and Methods

### 2.1. Study Design, Setting and Population

This study was designed as a retrospective observational cohort analysis comparing clinical outcomes and hospital resource utilization between patients diagnosed using rapid microbiological diagnostics and those diagnosed using conventional culture-based methods. A retrospective observational study was conducted over 5 years and 8 months (January 2015–August 2020) at the Department of Medical Microbiology and Immunology “Prof. Dr. Elissay Yanev” and the Innovative Diagnostic Methods Unit of the Research Institute, Medical University–Plovdiv, in collaboration with the Microbiology Laboratory of University Hospital “St. George,” Plovdiv. The analysis included 115 patients hospitalized at the Clinic of Anesthesiology and In-tensive Care of University Hospital “St. George.” Eligible patients had suspected or confirmed bloodstream infections (bacteremia or fungemia). This population represents a high-acuity cohort characterized by increased mortality risk, frequent need for organ support, prolonged hospitalization, and high resource utilization—a setting where rapid pathogen identification is expected to have the greatest potential clinical and economic impact. Patients were included if they met predefined inclusion and exclusion criteria. Baseline clinical characteristics were extracted from electronic medical records and included the primary reason for intensive care unit (ICU) admission (medical vs. surgical), presence of malignancy, immunocompromised status (including chemotherapy, hematologic disease, or long-term corticosteroid therapy), and presence of septic shock at admission. These variables were used to characterize the clinical complexity of the cohort and to contextualize subsequent cost analyses.

Inclusion Criteria: (1) Patients of any age or sex with ≥2 clinical indicators of systemic infection (e.g., fever >38 °C or <36 °C, leukocytosis >12 × 10^3^/µL or leukopenia <4 × 10^3^/µL, tachycardia >90 bpm, tachypnea >20 breaths/min, or PaCO_2_ < 32 mm Hg); (2) Blood cul-tures obtained before or ≥24 h after antibiotic administration. Exclusion Criteria: (1) Sampling without aseptic conditions; (2) Single-bottle blood culture in adults; (3) Only one or no clinical indicator of infection. Only microbiologically confirmed episodes of bacteremia or fungemia based on positive blood cultures were included in the final clinical and economic analysis. Rapid diagnostic results were promptly communicated to the ICU team. A consultative discussion between the clinical microbiologist and the attending physician addressed pathogen identification, resistance profile, and potential therapeutic implications, including escalation or de-escalation of antimicrobial therapy. This model was advisory, and final treatment decisions remained with the ICU team. No protocolized antimicrobial stewardship algorithm was implemented.

### 2.2. Definitions of Clinical Variables

Mortality was defined as: In-hospital death occurring during the same hospitalization episode in which bloodstream infection was diagnosed.

Empirical antibiotic therapy was defined as: Antimicrobial treatment initiated before microbiological identification of the causative pathogen and prior to availability of susceptibility testing results.

Targeted antibiotic therapy was defined as antimicrobial treatment adjusted or selected based on microbiological identification and, when available, antimicrobial susceptibility testing results.

Therapy modification was defined as any documented change in antimicrobial regimen following receipt of microbiological diagnostic results, including escalation, de-escalation, discontinuation, or switch to a different antimicrobial agent.

Antibiotic-days were calculated as the total number of calendar days during which a patient received at least one systemic antimicrobial agent.

### 2.3. Blood Culture Collection and Processing

Blood samples were collected by venipuncture under aseptic conditions and inoculated into aerobic and anaerobic bottles (adults) or aerobic bottles (pediatric patients) compatible with the BacT/ALERT 3D-60 automated blood culture system (bioMérieux, Craponne, France). Bottles were transported promptly to the microbiology laboratory and incubated for up to 7 days. Upon positivity signaling, immediate Gram staining was performed, followed by either conventional culture-based processing or rapid diagnostic testing according to laboratory workflow and platform availability during the study period. The BacT/ALERT system detects microbial growth via continuous colorimetric monitoring of CO_2_ production. All patients underwent primary bloodstream infection diagnostics using the BacT/ALERT 3D-60 automated blood culture system prior to any allocation to conventional or rapid downstream diagnostic workflows.

### 2.4. Conventional Culture-Based Diagnostic Workflow

For standard culture-based diagnostics, positive blood cultures underwent subculture on appropriate solid media (blood agar, chocolate agar, EMB agar, and Sabouraud dextrose agar as indicated) and incubation at 35 °C in ambient air or 5% CO_2_. Organism identification was performed using routine biochemical methods and automated identification systems (API and/or VITEK 2, bioMérieux). Antimicrobial susceptibility testing (AST) was conducted according to European Committee on Antimicrobial Susceptibility Testing (EUCAST) recommendations applicable to the respective year, using disk diffusion (Kirby-Bauer method), gradient MIC strips (Etest), automated MIC determination (VITEK 2), and broth microdilution when indicated. The conventional workflow required colony isolation before definitive identification and susceptibility reporting.

### 2.5. Rapid Diagnostic Methods

For the purpose of the present analysis, rapid diagnostic testing (RDT) refers to microbiological identification methods that enable earlier pathogen identification directly from positive blood culture material or through accelerated workflows compared with conventional culture-based identification requiring colony isolation. Rapid diagnostic testing was performed directly from positive blood culture bottles using one or more of the following platforms, depending on availability during the study period:

Multiplex PCR (FilmArray Blood Culture Identification Panel, BioFire Diagnostics/bioMérieux): The BCID panel was applied directly to positive blood culture material, enabling simultaneous identification of common Gram-positive bacteria, Gram-negative bacteria, Candida spp., and selected antimicrobial resistance genes (mecA, vanA/B, blaKPC, blaNDM, blaVIM, blaOXA, blaIMP). The analytical runtime was approximately 70 min.

Peptide Nucleic Acid Fluorescence In Situ Hybridization (PNA-FISH, QuickFISH BC, AdvanDx, Woburn, MA, USA): Fluorescent in situ hybridization was performed following Gram stain results for rapid identification of selected Gram-positive organisms (*Staphylococcus aureus*, *coagulase-negative staphylococci*, *Enterococcus faecalis*), Gram-negative bacteria (*Escherichia coli*, *Klebsiella pneumoniae*, *Pseudomonas aeruginosa*), and *Candida* species. The analytical runtime was approximately 40 min.

Matrix-Assisted Laser Desorption/Ionization Time-of-Flight Mass Spectrometry (MALDI-TOF MS, VITEK MS, bioMérieux): Identification was performed either from isolated colonies or via a direct-from-positive blood culture workflow using the VITEK MS Blood Culture Kit (research use only), incorporating selective lysis and centrifugation before spectral analysis and database matching for approximately 30 min per sample. MALDI-TOF identification performed after colony isolation was considered part of the conventional culture-based diagnostic workflow, whereas direct-from-positive blood culture MALDI workflows were classified within the rapid diagnostic testing strategy.

The identified pathogens included Gram-positive cocci (*Staphylococcus* spp., *Enterococcus* spp., *Streptococcus* spp.), Gram-negative bacilli (*Enterobacterales* including *Escherichia coli*, *Klebsiella* spp., *Enterobacter* spp., and non-fermenting organisms such as *Pseudomonas aeruginosa* and *Acinetobacter baumannii*), and yeasts (*Candida* spp.). Polymicrobial infections and contaminants were handled according to clinical and microbiological criteria.

All positive blood culture bottles underwent conventional culture-based processing and antimicrobial susceptibility testing, which served as the reference standard. When rapid diagnostic testing was available, it was performed directly from the same positive blood culture bottle in parallel with conventional processing. Therefore, rapid identification results were verified against conventional culture results. This parallel diagnostic approach allowed assessment of concordance between rapid and conventional methods under routine clinical conditions. The present study did not aim to formally calculate analytical sensitivity or specificity of the rapid diagnostic platforms, as their diagnostic performance has been extensively validated in prior multicenter studies. The primary objective was to evaluate clinical and economic impact.

### 2.6. Turnaround Time Definition

Turnaround time (TAT) was conceptually defined as the interval between blood culture collection and laboratory reporting of pathogen identification. However, due to incomplete timestamp documentation in the retrospective dataset, patient-level TAT values could not be reliably extracted or analyzed. Because standardized timestamps for organism identification and therapy modification were not consistently recorded in the retrospective dataset, process indicators such as time to identification and time to appropriate antimicrobial therapy could not be reliably analyzed.

### 2.7. Cost Components

Hospital Bed-Day Costs: The average cost per hospital bed-day was calculated using data from the accounting system of University Hospital “St. George”. The system allocates direct and indirect costs according to Bulgarian accounting standards. Direct costs include food for inpatients, medical supplies, blood and blood products, bioproducts, implants, and laboratory services. Indirect costs cover: utilities, maintenance, external services, depreciation, staff salaries, social security contributions, taxes, and other administrative expenses. All monthly expenses were summed to determine the average cost per bed-day for each patient, expressed separately as direct and indirect components.

Antibiotic Therapy Costs: Drug-related costs were extracted from direct hospital expenditures. For each patient, antibiotic costs were calculated based on the administered dose and unit price per day (“antibiotic-day”), using data from the hospital information system.

Diagnostic Test Costs: Laboratory costs were included in direct expenses, while microbiological test costs (rapid or culture-based) were also reported separately for clarity.

Clinical Pathways and Procedures: Costs for clinical pathways (CP) and clinical procedures (CPr) were calculated using official tariffs from the National Framework Agreements between the National Health Insurance Fund and the Bulgarian Medical Association (2017–2019). For procedures (CPr 3 and CPr 4), costs were estimated as a proportion of the total bed-days during which they were applied. Since these procedures are billed per 24 h and cannot overlap, their combined share (89.51%) was applied to individual patient data.

### 2.8. Group Allocation and Potential Selection Bias

No randomization procedure was applied, as this was a retrospective observational study. Allocation to the RDT or conventional culture group depended exclusively on laboratory platform availability and operational workflow at the time of blood culture positivity. The diagnostic approach applied to each positive blood culture depended on laboratory platform availability and operational workflow at the time of positivity during the study period. Rapid diagnostic platforms were implemented progressively between 2015 and 2020, and their use was determined by availability rather than predefined clinical criteria. No formal protocol existed to prioritize rapid diagnostic testing based on disease severity, comorbidities, or anticipated prognosis. Diagnostic modality was therefore independent of predefined patient-level selection criteria. To explore potential imbalance between groups, secondary analyses were performed stratifying patients according to clinical severity category and outcome variables. These analyses did not demonstrate systematic differences, suggesting intentional preferential allocation. Given the observational design, residual confounding cannot be completely excluded and is acknowledged as a limitation.

### 2.9. Statistical Methods

Quantitative variables with a normal distribution were expressed as mean ± standard deviation (SD). Variables without normal distribution were presented as median and interquartile range (25th–75th percentiles) and analyzed using the Mann–Whitney U. Qualitative variables were summarized as frequencies and percentages (*n*, %). Differences between two proportions were tested using the z-test, while deviations between theoretical and observed distributions were assessed with the chi-square test. A *p*-value < 0.05 was considered statistically significant. Data were processed using IBM SPSS Statistics for Windows, version 26.0 (Armonk, NY, USA: IBM Corp.)

## 3. Results

### 3.1. Patient Demographics and Clinical Characteristics

A total of 115 patients were included in the study, 69.6% male, with a median age of 52 years (33, 62). No statistically significant differences were observed between the two diagnostic groups regarding age (U = 1288, *p* = 0.298) or sex (z = 1.1, *p* = 0.267). Baseline characteristics are summarized in Table 1. The spectrum and relative frequency of microorganisms identified in bloodstream infections are presented in Table 2. A total of 115 patients with confirmed bloodstream infections were included in the analysis. Overall, 118 microorganisms were isolated from positive blood cultures, reflecting the presence of mixed infections in a small number of cases. Coagulase-negative *Staphylococcus* species represented the most frequently detected group (13.9%). To minimize the possibility of contamination, coagulase-negative staphylococci were considered clinically significant only when confirmed in three separate blood culture sets. Gram-negative pathogens such as *Escherichia coli*, *Acinetobacter baumannii*, and *Pseudomonas aeruginosa* were also commonly identified, while several fungal isolates, predominantly *Candida* species, were observed.

### 3.2. Length of Stay and Antibiotic Therapy

The median hospital stay was 20 days (12, 35) in patients tested with rapid diagnostics and 16 days (10, 38) in those tested by standard culture (U = 1351, *p* = 0.505). The number of antibiotic-days for empirical therapy was similar between groups (U = 1456, *p* = 0.967). However, the duration of targeted therapy was approximately 50% longer in the rapid-test group (median 12 days) than in the culture group (median 6 days), though the difference was not statistically significant (U = 1160, *p* = 0.070). Therapy was modified following diagnostic results in 63.6% of patients with rapid tests and 57.9% of those with culture tests (z = 0.6, *p* = 0.551). Mortality rates were comparable between groups (54.5% vs. 55.3%, z = 0.1, *p* = 0.942). Rapid testing was not associated with a statistically significant reduction in mortality risk (OR = 0.97; 95% CI 0.45–2.12; *p* = 0.942).

### 3.3. Differences Associated with the Culture-Based Methods

Table 3 summarizes cumulative hospital resource utilization associated with each diagnostic strategy, including total bed-days, antibiotic-days, and overall hospital expenditures. Due to the limited number of patients in this subgroup, the results are presented descriptively to illustrate patterns of hospital resource utilization. Descriptive comparison of cumulative resource utilization suggested numerically: longer average hospital stay (34%), longer empirical antibiotic therapy (6%), and approximately 35% higher direct, indirect, and clinical procedure costs in the culture group. For all other indicators, the differences were negative, indicating that higher values were observed among patients diagnosed with rapid tests—notably a 50% longer duration of targeted antibiotic therapy, resulting in an 18% longer total treatment duration and a 40% increase in the ratio of antibiotic-days to hospital bed-days. These differences were reflected in average antibiotic treatment costs, most prominently in the targeted therapy component, which was 96% higher among patients diagnosed using rapid methods. Despite the higher antibiotic expenses, the savings from hospital bed-days (direct non-drug and indirect costs) were substantial, resulting in an overall lower total financial expenditure for the rapid testing group.

## 4. Discussion

Rapid diagnostic testing (RDT) for bloodstream infections in critically ill patients, particularly when implemented alongside antimicrobial stewardship programs (ASPs), has been associated with improved clinical and economic outcomes compared with conventional culture methods. Meta-analytic evidence suggests that mortality reduction becomes significant primarily when rapid diagnostics are combined with stewardship interventions (odds ratio 0.72 vs. blood culture alone; odds ratio 0.78 vs. blood culture plus stewardship) [3]. These benefits are generally not observed when rapid diagnostics are used without stewardship support or when stewardship is applied to conventional cultures alone [2,3].

Several of the observed differences between diagnostic strategies did not reach statistical significance; therefore, the findings should be interpreted cautiously and considered hypothesis-generating rather than definitive evidence of superiority.

In our real-world cohort from a Bulgarian tertiary hospital, rapid diagnostics produced clinical outcomes comparable to conventional culture-based methods, with no statistically significant differences in mortality, therapy modification, or total hospital stay. The relatively high mortality rate observed in this cohort (~55%) reflects the severity of illness among critically ill ICU patients with bloodstream infections and is consistent with mortality rates reported in severe sepsis and septic shock populations, which often range between 30% and 60% in intensive care settings [15,16,17]. Patients in the rapid-test group received longer targeted antibiotic therapy, which may reflect differences in antimicrobial management following microbiological identification, although this study did not formally evaluate time to therapy optimization. These observations are broadly consistent with previously published international studies suggesting that rapid diagnostics may facilitate earlier optimization of antimicrobial therapy compared with conventional cultures [3,18]. Current clinical guidelines also emphasize the importance of timely communication of diagnostic results to support faster treatment adjustments and improved antimicrobial stewardship practices [1,2,18,19].

Although randomized trials and meta-analyses demonstrate variable effects on mortality, they consistently indicate that rapid diagnostics facilitate earlier therapy modification. For example, in Gram-negative bacteremia, the median time to first antibiotic change has been reported as 8.6 h with rapid testing versus 14.9 h with standard culture, while escalation for resistant infections decreased from 61.7 to 18.4 h [6]. A systematic review evaluating rapid versus standard antimicrobial susceptibility testing in bloodstream infections similarly reported that rapid diagnostic approaches can shorten the time to appropriate antimicrobial therapy, although evidence for improvements in mortality and other clinical outcomes remains variable across studies [20]. Real-world studies similarly report increased proportions of patients receiving optimized antimicrobial therapy within 24 h of a positive culture result [21]. In our analysis, therapy modification occurred in 63.6% of patients in the rapid-test group compared with 57.9% in the culture group, demonstrating a similar directional trend, although the difference did not reach statistical significance. Importantly, improvements in mortality and other patient-centered outcomes remain less consistently demonstrated in randomized trials, with several meta-analyses reporting minimal differences in mortality or time to discharge [22]. Although our study population was not randomized and patient groups may differ in clinical characteristics, the analysis provides a real-world comparison of clinical outcomes and hospital resource utilization between rapid diagnostic testing and conventional microbiological diagnostics.

From a resource-utilization perspective, rapid diagnostics in our study were associated with higher diagnostic and clinical pathway costs due to the use of advanced technologies. However, patterns of hospital resource use suggested potential economic differences between diagnostic strategies. Although the median length of stay did not differ significantly between groups, differences in total hospital costs were primarily driven by variations in procedural and hospitalization expenditures rather than statistically significant differences in length of stay. Previous studies have reported that implementation of rapid platforms such as Accelerate Pheno can reduce median length of stay and antibiotic days of therapy [18]. Economic modeling studies also indicate that molecular rapid diagnostics combined with stewardship interventions may reduce overall hospital costs and antimicrobial exposure [1,5]. Recent randomized trial evidence also highlights the economic implications of rapid molecular diagnostics in critical care settings. The multicentre INHALE study demonstrated that ICU-based syndromic PCR testing may improve antimicrobial management and has the potential to be cost-effective when implemented within structured stewardship pathways [23].

The broader economic literature consistently emphasizes that the value of rapid diagnostics depends strongly on their integration within antimicrobial stewardship programs. Studies such as those by Pliakos et al. demonstrate that rapid diagnostics combined with stewardship can produce substantial cost savings and improved quality-adjusted life years, whereas isolated implementation of molecular diagnostics yields more variable benefits [1]. Other studies similarly report that earlier therapy optimization and antibiotic modification occur more frequently when stewardship actively guides interpretation of rapid diagnostic results [18].

Effective implementation of rapid diagnostics in critically ill patients requires coordinated laboratory workflows, clinician training, and multidisciplinary collaboration, including clinical bioinformatics expertise [24,25,26]. The clinical and economic impact of these technologies depends largely on how rapidly diagnostic information is integrated into treatment decisions. Although the present study did not demonstrate statistically significant reductions in mortality or hospital stay, it provides real-world evidence suggesting that rapid diagnostics may support more targeted antimicrobial therapy and influence patterns of hospital resource utilization. Further research is needed to standardize testing protocols and to better define the cost-effectiveness of rapid diagnostics in specific intensive care unit populations [2,10,25,26].

Given the heterogeneity of rapid diagnostic platforms used and the absence of standardized diagnostic process indicators in the retrospective dataset, these findings should be interpreted as exploratory real-world observations rather than definitive evidence of diagnostic superiority.

### Limitations

This study has several limitations. First, its retrospective single-center design may limit the generalizability of the findings to other healthcare settings. The sample size, particularly within subgroups of critically ill patients, was relatively small and may have reduced the statistical power to detect modest differences in clinical outcomes; therefore, the results should be interpreted as exploratory and descriptive rather than definitive comparative evidence. In addition, multivariable regression analyses were not performed because several key clinical covariates—such as standardized severity scores, detailed comorbidity measures, infection source classification, and organ support parameters—were not consistently available in the retrospective dataset. Consequently, the analyses were limited to unadjusted comparisons and should be interpreted cautiously.

Second, the economic analysis relied on hospital accounting data, which may not capture all downstream or societal costs, such as readmissions or long-term healthcare expenditures.

Third, clinical outcomes were based on routinely collected clinical data and may have been influenced by patient severity, comorbidities, and variations in antimicrobial management that were not fully controlled for in this analysis. Detailed classification of the primary clinical conditions leading to ICU admission was not consistently available in the retrospective dataset, which limited the possibility of stratifying outcomes according to underlying disease categories. In addition, the analysis did not stratify outcomes by specific pathogens or underlying clinical diagnoses, which may influence antimicrobial treatment strategies and associated healthcare costs. Detailed antimicrobial resistance profiles were also not analyzed, as susceptibility testing data were not systematically standardized across diagnostic pathways in the retrospective dataset.

Fourth, the study did not include a formal incremental cost-effectiveness analysis that would allow estimation of cost per health outcome gained. Similarly, the analysis did not incorporate adjusted cost estimates or sensitivity analyses; therefore, the reported cost differences should be interpreted as descriptive indicators of hospital resource utilization rather than definitive estimates of cost-effectiveness.

Finally, the study did not evaluate key diagnostic process indicators such as turnaround time (TAT), time to pathogen identification, or time to appropriate therapy because standardized timestamp data were not consistently available across the retrospective study period, preventing reliable reconstruction of diagnostic timelines. Consequently, the analysis focused on downstream clinical and economic outcomes rather than laboratory process metrics.

Future multicenter prospective studies are warranted to validate these findings and further assess the clinical and economic impact of rapid microbiological diagnostics in Bulgaria. Given the observational design and modest sample size of the cohort, the present findings should be interpreted cautiously and primarily as hypothesis-generating.

## 5. Conclusions

Rapid microbiological diagnostics for bloodstream infections, particularly when implemented alongside antimicrobial stewardship programs, may support clinical decision-making in critically ill patients. In this real-world Bulgarian cohort, rapid diagnostic testing produced clinical outcomes comparable to conventional culture-based diagnostics, with no statistically significant differences in mortality, therapy modification rates, or overall hospital stay. Patients in the rapid-testing group demonstrated longer targeted antibiotic therapy, which may reflect differences in antimicrobial management following microbiological identification.

Although rapid diagnostics were associated with higher upfront testing costs, the analysis suggested differences in hospital resource utilization and cost patterns between diagnostic approaches. Given the retrospective design, lack of multivariable adjustment, and limited sample size in some analyses, these findings should be interpreted cautiously and considered hypothesis-generating rather than definitive evidence of economic advantage.

Overall, the study provides real-world data from a Central and Eastern European tertiary-care setting and suggests that rapid microbiological diagnostics, when integrated into antimicrobial stewardship frameworks, may influence antimicrobial management and hospital resource use. Further multicenter prospective studies with larger cohorts and more comprehensive economic evaluation are needed to better define their clinical and economic impact.

## Figures and Tables

**Table 1:** Comparison of patient characteristics by diagnostic method—rapid diagnostic tests versus standard culture-based testing.

Variable	Rapid Tests (*n* = 77)	Standard Culture (*n* = 38)	*p*-Value
**Demographic characteristics**			
Male, *n* (%)	51 (66.20)	29 (76.32)	0.267 ^1^
Age (years), median; 25th, 75th percentiles	54; 39, 65	51; 30, 60	0.298 ^2^
**Clinical data**			
Length of stay (days), median; 25th, 75th percentiles	20; 12, 35	16; 10, 31	0.505 ^2^
Antibiotic days—empirical therapy (days), median; 25th, 75th percentiles	13; 7, 25	14; 8, 20	0.967 ^2^
Antibiotic days—targeted therapy (days), median; 25th, 75th percentiles	12; 5, 28	6; 1, 18	0.070 ^2^
Antibiotic days—total (days), median; 25th, 75th percentiles	31; 14, 49	23; 12, 32	0.111 ^2^
Therapy modification after diagnostic result—yes, *n* (%)	49 (63.64)	22 (57.89)	0.551 ^1^
**Changes in therapy after diagnostic test, *n* (%)**			
Died before result	14 (18.18)	9 (23.68)	0.488 ^1^
Continued same antibiotic therapy	14 (18.18)	7 (18.42)	0.975 ^1^
Discontinuation of antibiotic therapy	1 (1.30)	0 (0.00)	–
Change in antibiotic therapy	48 (62.34)	22 (57.89)	0.646 ^1^
Mortality, *n* (%)	42 (54.54)	21 (55.26)	0.942 ^1^
**Costs**			
Direct costs (BGN), median; 25th, 75th percentiles	2319.40; 1333.66, 4058.95	1855.52; 1159.70, 3595.07	0.505 ^2^
Indirect costs (BGN), median; 25th, 75th percentiles	19,388.80; 11,148.56, 33,930.40	15,511.04; 9694.40, 30,052.64	0.505 ^2^
Cost of clinical pathways (BGN), median; 25th, 75th percentiles	3252.00; 1546.50, 4290.00	2442.00; 839.00, 3267.00	**0.030** ^2^
Cost of clinical procedures (BGN), median; 25th, 75th percentiles	10,444.06; 6005.33, 18,277.10	8355.25; 5222.03, 16,188.29	0.505 ^2^
Cost of diagnostic tests (BGN), median; 25th, 75th percentiles	55.00; 2.00, 400.00	38.00; 38.00, 38.00	**0.002** ^2^
Total antibiotic therapy cost (BGN), median; 25th, 75th percentiles	779.78; 294.05, 1608.68	589.80; 204.96, 1401.57	0.241 ^2^
Antibiotic therapy cost—empirical (BGN), median; 25th, 75th percentiles	342.81; 95.52, 652.74	236.07; 98.49, 531.75	0.293 ^2^
Antibiotic therapy cost—targeted (BGN), median; 25th, 75th percentiles	338.90; 26.09, 965.63	256.41; 22.12, 748.80	0.498 ^2^

^1^ z-test for comparison of two proportions; ^2^ Mann-Whitney U test.

**Table 2:** Distribution of microorganisms identified in bloodstream infections in the study population (*n* = 115).

Microorganism	Count (*n*)	Percentage (%)
*Coagulase-negative Staphylococcus*	16	13.91
*Escherichia coli*	13	11.30
*Acinetobacter baumannii*	12	10.43
*Pseudomonas aeruginosa*	12	10.43
*Candida albicans*	11	9.57
*Klebsiella pneumoniae*	10	8.70
*Candida parapsilosis*	7	6.09
*Enterococcus faecalis*	7	6.09
*Enterobacter cloacae complex*	7	6.09
*Enterococcus faecium*	6	5.22
*Candida lusitaniae*	4	3.48
*Streptococcus anginosus*	3	2.61
*Burkholderia cepacia complex*	3	2.61
*Staphylococcus aureus*	2	1.74
*Serratia marcescens*	2	1.74
*Proteus mirabilis*	2	1.74
*Klebsiella oxytoca*	1	0.87

**Table 3:** Cumulative hospital resource utilization and financial impact associated with rapid versus conventional microbiological diagnostics in critically ill patients with antibiotic therapy modification.

	Rapid Tests (*n* = 20)	Standard Culture (*n* = 8)		
Hospital stay (bed-days)	453	277		
Antibiotic-days—total	769	261		
Antibiotic-days—empirical therapy	305	136		
Antibiotic-days—targeted therapy	464	125		
Ratio of antibiotic-days to bed-days	48	8		
Direct costs (BGN)	5253.41	32,123.69		
Indirect costs (BGN)	43,916.32	26,853.88		
Clinical pathway costs (BGN)	74,88.00	26,884.00		
Clinical procedure costs (BGN)	23,655.87	14,465.18		
Total antibiotic costs (BGN)	3154.77	7341.66		
Antibiotic costs—empirical therapy (BGN)	11,113.79	3174.54		
Antibiotic costs—targeted therapy (BGN)	20,433.98	4167.12		
**Differences associated with the Culture-Based Methods**			*** n ***	** % **
Average hospital stay (bed-days)	23	35	12	34
Average total treatment duration (antibiotic-days)	39	33	−6	−18
Average empirical therapy duration (antibiotic-days)	16	17	1	6
Average targeted therapy duration (antibiotic-days)	24	16	−8	−50
Average ratio of antibiotic-days to bed-days	2.4	1	−1.4	−140
Average direct costs (BGN)	2626.72	4015.46	1388.74	35
Average indirect costs (BGN)	21,957.82	33,566.86	11,609.04	35
Average clinical pathway costs (BGN)	3743.4	3360.5	−382.9	−11
Average clinical procedure costs (BGN)	11,827.89	18,081.27	6253.38	35
Average total antibiotic costs (BGN)	1577.39	917.71	−659.68	−72
Average empirical antibiotic costs (BGN)	555.69	396.82	−158.87	−40
Average targeted antibiotic costs (BGN)	1021.7	520.89	−500.81	−96

## Data Availability

Research data can be obtained upon request.

## Abbreviations

The following abbreviations are used in this manuscript:

ASPs	Antimicrobial stewardship programs
BSIs	Bloodstream infections
CP	Clinical pathways
CPr	Clinical procedures
ICU	Intensive care unit
mRDTs	Molecular rapid diagnostic tests
RDTs	Rapid diagnostic tests
SD	Standard deviation
